# Total 25-Hydroxyvitamin D Is an Independent Marker of Left Ventricular Ejection Fraction in Heart Failure with Reduced and Mildly Reduced Ejection Fraction

**DOI:** 10.3390/biom13111578

**Published:** 2023-10-26

**Authors:** Timea Magdolna Szabo, Előd Ernő Nagy, Ádám Kirchmaier, Erhard Heidenhoffer, Hunor-László Gábor-Kelemen, Marius Frăsineanu, Judit Cseke, Márta Germán-Salló, Attila Frigy

**Affiliations:** 1 Doctoral School of Medicine and Pharmacy, George Emil Palade University of Medicine, Pharmacy, Science and Technology of Târgu Mureș, 540142 Târgu Mureș, Romania; 2 Department of Cardiology, Clinical County Hospital Mureș, 540103 Târgu Mureș, Romania; kirchmaier.adam@gmail.com (Á.K.); heiden.erhard@gmail.com (E.H.); gaborhunor2014@gmail.com (H.-L.G.-K.); frasineanu.marius@gmail.com (M.F.); csekejudiit@gmail.com (J.C.); attila.frigy@umfst.ro (A.F.); 3 Department of Biochemistry and Environmental Chemistry, George Emil Palade University of Medicine, Pharmacy, Science and Technology of Târgu Mureș, 540142 Târgu Mureș, Romania; elod.nagy@umfst.ro; 4 Laboratory of Medical Analysis, Clinical County Hospital Mureș, 540394 Târgu Mureș, Romania; 5 Department of Internal Medicine III, George Emil Palade University of Medicine, Pharmacy, Science and Technology of Târgu Mureș, 540142 Târgu Mureș, Romania; marta.german-sallo@umfst.ro; 6 Department of Internal Medicine IV, George Emil Palade University of Medicine, Pharmacy, Science and Technology of Târgu Mureș, 540142 Târgu Mureș, Romania

**Keywords:** heart failure, heart failure with reduced ejection fraction, heart failure with mildly reduced ejection fraction, vitamin D deficiency, serum albumin, serum uric acid

## Abstract

Vitamin D emerged as an important prognostic biomarker in heart failure (HF), with currently highly debated therapeutic implications. Several trials on vitamin D supplementation in HF showed improvements in left ventricular (LV) remodeling and function and health-related quality of life (HRQoL), which did not translate into mid- to long-term beneficial effects regarding physical performance and mortality. We addressed total 25-hydroxyvitamin D (25(OH)D), serum albumin, and uric acid (UA) levels, focusing mainly on vitamin D deficiency, as potential markers of LV systolic dysfunction in HF with reduced and mildly reduced ejection fraction (HFrEF, HFmrEF). Seventy patients with LVEF < 50% were comprehensively evaluated using ECG, echocardiography, lung ultrasound (LUS), blood sampling, and the six-minute walk test (6MWT). HRQoL was also assessed using the Minnesota Living with Heart Failure Questionnaire (MLHFQ). Statistically significant positive correlations were found between LVEF, 25(OH)D, serum UA, and albumin, respectively (*p* = 0.008, *p* = 0.009, and *p* = 0.001). Serum UA (7.4 ± 2.4 vs. 5.7 ± 2.1, *p* = 0.005), NT-proBNP levels (1090.4 (675.2–2664.9) vs. 759.0 (260.3–1474.8), *p* = 0.034), and MLHFQ scores (21.0 (14.0–47.0) vs. 14.5 (4.5–25.5), *p* = 0.012) were significantly higher, whereas 25(OH)D concentrations (17.6 (15.1–28.2) vs. 22.7 (19.5–33.8), *p* = 0.010) were lower in subjects with severely reduced LVEF. Also, 25(OH)D was independently associated with LVEF in univariate and multiple regression analysis, maintaining its significance even after adjusting for confounders such as age, NT-proBNP, the presence of chronic coronary syndrome, hypertension, and anemia. According to our current findings, 25(OH)D is closely associated with LVEF, further supporting the need to establish correct vitamin D supplementation schemes and dietary interventions in HF. The changes in LVEF, 25(OH)D, serum UA, and albumin levels in HFrEF and HFmrEF indicate a similar pathophysiological background.

## 1. Introduction

Vitamin D is a pleiotropic hormone showing substantial cardiovascular involvement beyond its regulatory effects on calcium and phosphate homeostasis. Vitamin D deficiency is frequently associated with cardiovascular disease [1] and is increasingly prevalent in HF [2]. Patients with HF are prone to develop malnutrition and subsequent vitamin D deficiency due to intestinal epithelial barrier dysfunction, systemic venous congestion, low intake (because of dietary restrictions), treatment-associated macronutrient and micronutrient depletion, and comorbidity-related conditions [3]. Also, elevated levels of pro-inflammatory cytokines in HF are linked to chronic hypercatabolic syndrome [3]. Although the vitamin D and parathyroid hormone status of patients did not predict the development of HF in the PREVEND population after adding potential confounders [4], emerging evidence links the parameters of calcium and phosphate metabolism to the progression of HF [5].

25-hydroxyvitamin D (25(OH)D) is an abundant circulating metabolite and a reliable indicator of vitamin D status [6]. Less than 1% is free, as it binds mainly (85%) to the vitamin D-binding protein (DBP), and with lower affinity to albumin (15%) [7]. 1,25-dihydroxyvitamin D_3_ (1,25(OH)_2_D_3_), the biologically active form of vitamin D, exerts its genomic and rapid, non-genomic effects via vitamin D receptors (VDRs) [8]. Both experimental and clinical trials highlight the important role played by vitamin D deficiency in the activation of the renin–angiotensin–aldosterone system (RAAS), the phenotypic transformation of cardiac fibroblasts, and shift of balance in myocardial extracellular matrix turnover, with subsequent cardiac remodeling and dysfunction [5]. Also, patients with end-stage HF, who underwent heart transplant, showed low expression of VDRs and ensuing Th2-biased inflammation in the failing heart [9]. 1,25(OH)_2_D proved to mediate the fine balance between Treg and Th17 cells, promoting an anti-inflammatory response in chronic HF [10]. Moreover, vitamin D supplementation in HF reduced C-reactive protein (CRP) levels [3]. Patients with HF, who received a weekly high dose of vitamin D (50,000 IU) for 2 months, showed reversed LV remodeling—improved LV end-diastolic volumes (LVEDV), LV ejection fraction (LVEF), and also a significant increase in serum albumin levels [11].

HF induces a number of compensatory mechanisms, and the two most important are the activation of the sympathetic nervous system and the RAAS, both having deleterious long-term hemodynamic consequences. Low-grade systemic inflammation and oxidative stress are also key contributors to the development and progression of HF [12]. Chronic inflammation can be described as a prolonged, rather blunted inflammatory response to different endogenous and exogenous stimuli, also modulating acute-phase protein (APP) synthesis [13]. Albumin, a well-established prognostic marker in HF [14], is a negative APP, as its levels decrease with inflammation [13]. Furthermore, hypoalbuminemia is often the expression of the magnitude of biological stress triggered by inflammation, rather than inadequate nutritional intake [15]. Considering the fact that albumin is the main determinant of colloid osmotic pressure, hypoalbuminemia is a frequent cause of impaired fluid distribution and build-up (edema) [16]. Also, serum albumin has strong plasma thiol-dependent antioxidant activity [17].

Uric acid (UA), the end product of human purine metabolism, proved to exhibit both antioxidant and pro-oxidant effects in a concentration-dependent manner [18]. Over the last two decades, serum UA resurfaced as a valuable prognostic biomarker in HF [19,20,21], mainly because of its major involvement in oxidative stress, endothelial dysfunction, and local inflammation [22]. Xanthine-oxidase (XO), the key enzyme in the production of UA, also responsible for generating reactive oxygen species (ROS), is upregulated in patients with HF and hyperuricemia, contributing to adverse outcomes [22]. Moreover, serum UA, by further activating the RAAS, showed positive correlations with echocardiographic markers of LV remodeling and dysfunction [23,24].

The aim of our study was to assess the relationship between established biomarkers (vitamin D, serum albumin, UA) in HF and LV systolic function assessed using echocardiography in patients with HFrEF and HFmrEF. We analyzed whether the aforementioned circulating markers are associated with HF severity and whether these can predict LVEF in multiple logistic regression models.

## 2. Materials and Methods

### 2.1. Participants

Seventy patients with LV systolic dysfunction were included in a prospective, observational, single-center study between January 2022 and June 2023 in Targu Mures, Romania. Participants were recruited either from ambulatory care or were inpatients, and evaluated before discharge. We applied the following inclusion criteria: existing HFrEF or HFmrEF in conformity with the 2021 ESC guidelines [25], New York Heart Association (NYHA) class I–III, and overall clinical stability. Patients were excluded from the study if they showed signs and/or symptoms of infection, had diagnosed cancer, autoimmune disorders, liver disease, and poor kidney function (with estimated glomerular filtration rate (eGFR) < 20 mL/min/1.73 m^2^ using the Chronic Kidney Disease Epidemiology Collaboration equation [26]) [12]. Routine demographic, clinical, laboratory, and ultrasound (heart and lung) data were collected from each participant. The 6MWT distance was measured for the assessment of functional capacity, and further risk stratification. HRQoL was also evaluated using the Minnesota Living with Heart Failure Questionnaire (MLHFQ). The study was conducted in accordance with the Declaration of Helsinki; the Ethics Committee approved the study protocol (7716/02.07.2021, Clinical County Hospital Mureș; 2281/13.04.2023, George Emil Palade University of Medicine, Pharmacy, Science and Technology of Targu Mures), and all patients provided written informed consent before enrollment.

### 2.2. Ultrasound Evaluation

#### 2.2.1. Transthoracic Cardiac Ultrasound

Echocardiography was performed using a Philips Epiq7 ultrasound machine (Philips Ultrasound, Inc., Bothell, WA, USA) and a Philips X5-1 xMATRIX array transducer (1–5 MHz; Philips Ultrasound, Inc., Bothell, WA, USA). Clinical recommendations for multimodality cardiovascular imaging were applied [27,28,29]. LVEF was assessed using the biplane method of disks, whereas LV global longitudinal strain (LVGLS) was measured using the averaged data from all three standard apical views. LV and left atrial (LA) volumes were normalized by body surface area (BSA), which was determined with the Mosteller formula. Pulsed-wave tissue Doppler-derived E/e’ ratio was obtained by averaging e’ velocities acquired from the medial and lateral mitral annulus.

#### 2.2.2. Lung Ultrasound

The lung ultrasound (LUS) protocol consisted of an eight-zone evaluation, four on each hemithorax. Semi-recumbent positioning of patients facilitated the assessment of B-lines. The same cardiac probe was used for lung imaging as well, and depth was adjusted individually. The following threshold for abnormality (in terms of lung congestion) was applied: ≥3 B-lines per zone [30].

### 2.3. Laboratory Analysis

#### 2.3.1. Methodology

Blood samples were collected after twelve-hour fasting (Becton-Dickinson Vacutainer Systems, Wokingham, Berkshire, UK), and centrifuged for 10 min at 3000 rpm. Laboratory tests were performed, including total-, LDL-, and HDL-cholesterol, serum triglycerides, UA, creatinine, albumin, serum iron, ferritin, and CRP using an Architect C4000 (Abbott Laboratories, Diagnostic Division, Abbott Park, IL, USA); plasma fibrinogen was assessed with Sysmex CA-1500 (Sysmex Corporation, Kobe, Japan). N-terminal pro-B-type natriuretic peptide (NT-proBNP) concentrations were measured using an electrochemiluminescent method on an Elecsys 2010 (Roche Diagnostics International, Rotkreuz, Switzerland). Complete blood count was assessed using tripotassium ethylenediaminetetraacetic acid precoated tubes on a Mindray BC6000 (Mindray Global, Shenzhen, China).

#### 2.3.2. Serum Total 25-Hydroxyvitamin D

Quantitative determination ofthe total 25(OH)D in patients with HF was conducted using a competitive electrochemiluminescence protein binding assay on a Mindray CL-900i Chemiluminescence Immunoassay Analyzer (Mindray Bio-Medical Electronics Co., Ltd., Shenzhen, China). Patients were divided into tertiles based on serum 25(OH)D levels (first, 7.32–16.67 ng/mL; second, 17.06–26.38 ng/mL; third, 27.03–57.20 ng/mL). None of the participants were taking vitamin D supplements in the two months prior to the evaluation. Dietary vitamin D intake from food sources and sun exposure were not assessed. The reference range for this assay was 30–100 ng/mL.

### 2.4. Statistical Analysis

The Kolmogorov–Smirnov and/or Shapiro–Wilk normality tests were used for the assessment of data distribution. Paired t-test and Pearson’s correlation were performed for variables showing normal distribution. Data following non-Gaussian distribution were processed using the Mann–Whitney U test and Spearman’s rank correlation analysis. Categorical variables were quantified for absolute and relative frequency, and 2 × 2 contingency tables were analyzed with the χ^2^ test. 25(OH)D concentrations were log-transformed for analysis to improve normal distribution. *p*-values < 0.05 were regarded as statistically significant. Microsoft Excel 2016 (Microsoft Corporation, Redmond, WA, USA) and GraphPad Prism 9.5.0 (GraphPad Software LLC., San Diego, CA, USA) were used for data curation and processing.

## 3. Results

### 3.1. Study Group Characteristics—Overall Group, HFrEF vs. HFmrEF

Seventy patients (51 male, 72.85%) with a mean age of 66 ± 11 years were included in the study. Table 1 summarizes the basic characteristics and conditions at the time of recruitment; it also highlights differences between the studied HF groups—HFrEF and HFmrEF. Significantly more patients with HFrEF were hospitalized at the time of enrollment (46 vs. 24, *p* = 0.019). Patients with HFrEF had higher body mass index (BMI) (*p* = 0.025), LA volume index (LAVI) (*p* = 0.032), LVEDV index (LVEDVI) (*p* < 0.0001), LV end-systolic volume index (LVESVI) (*p* < 0.0001), lower stroke volume index (SVI) (*p* = 0.0002), longer-lasting heart disease (*p* = 0.041), compared to patients with HFmrEF. Also, study participants with HFmrEF showed better quality of life (*p* = 0.012). Serum UAd and NT-proBNP concentrations were significantly increased (*p* = 0.005, *p* = 0.034), whereas 25(OH)D levels were significantly decreased (*p* = 0.010) in HFrEF. Loop diuretic therapy was more frequently prescribed to patients with HFrEF. None of the participants took serum UA-lowering medication at the time of evaluation.

### 3.2. Correlations for 25(OH)D

**Figure 1:**
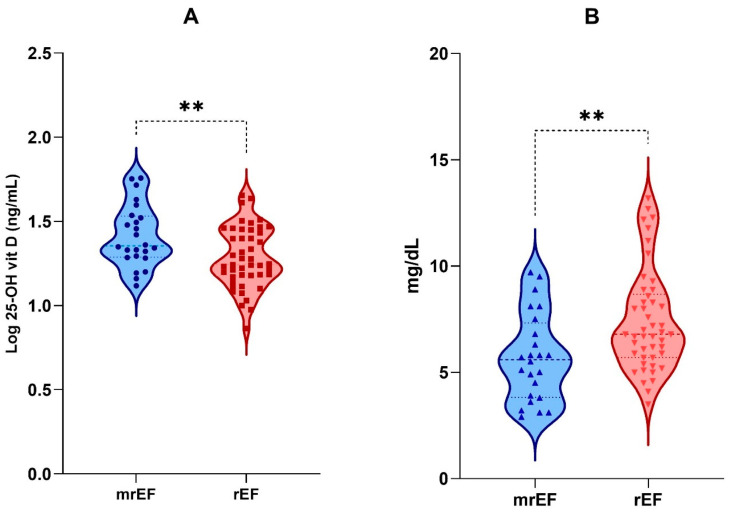
Violin plots depicting distributions for (**A**) log-transformed 25(OH)D and (**B**) serum uric acid in HFrEF and HFmrEF. The medians and the quartile values are indicated by the dotted lines. ** *p*-value < 0.01.

Both log 25(OH)D and serum UA showed significant differences and an inverse relationship in the two study groups (*p* < 0.01) (Figure 1).

Also, when considering 25(OH)D levels in all patients, significant positive correlations were found with diastolic blood pressure values (r = 0.27, *p* = 0.023), LVEF (r = 0.31, *p* = 0.008), platelet count (r = 0.25, *p* = 0.032), serum albumin (r = 0.37, *p* = 0.001), and HDL-cholesterol concentrations (r = 0.23, *p* = 0.048). Statistically significant negative associations were reported between serum 25(OH)D and UA levels (r = −0.29, *p* = 0.014), LVESVI (r = −0.25, *p* = 0.033), and LVGLS (r = −0.30, *p* = 0.012). No significant relationship was found connecting 25(OH)D to CRP (r = −0.19, *p* = 0.110), 6MWT distance (r = 0.19, *p* = 0.098), or NYHA class (r = −0.11, *p* = 0.348). Patients with 25(OH)D levels in the first tertile vs. the third tertile associated type II diabetes mellitus (DM) more frequently, but it did not reach statistical significance (10 patients vs. 4 patients; OR 3.65, 95% CI 0.94–14.20, *p* = 0.061).

### 3.3. Correlations for LVEF in HF

Table 2 shows the main correlations for LVEF in the study cohort. Significant negative correlations were observed between LVEF and patient length of stay (*p* = 0.003), BMI (*p* = 0.006), and E/e’ ratio (*p* = 0.037). LVEF also showed strong negative associations with NT-proBNP (*p* = 0.029), serum UA levels (*p* = 0.009), GGT activity (*p* = 0.006), and thyroid-stimulating hormone (TSH) concentrations (*p* = 0.040). Interestingly, LVEF was not linked to 6MWT distance, age, duration of heart disease, or duration of HF. Significant positive correlations were found between LVEF and both components of blood pressure—systolic (*p* = 0.003) and diastolic blood pressure (*p* = 0.006), and also serum 25(OH)D levels (*p* = 0.008).

### 3.4. Predictors of LVEF in HF

Multivariate linear regression models (A and B) were constructed in the overall study population for the prediction of LVEF in HF (Table 3). Log 25(OH)D remained significant in both models (*p* = 0.036 and *p* = 0.030, respectively). In the second model, log 25(OH)D maintained its predictive value, even after adjusting for the presence of chronic coronary syndrome (CCS).

### 3.5. Predictors of HFrEF

Nonlinear logistic regression models (C and D) were also set up to predict LV systolic dysfunction (Table 4). In the first model, when adjusted for BMI tertiles, serum UA tertiles, albumin tertiles, GGT tertiles, the presence of hypertension, atrial fibrillation, and anemia, the odds ratio conferred by log 25(OH)D in the lower tertile was 4.02 (1.05–15.42, 95% CI), and log 25(OH)D remained a significant independent determinant of HFrEF (*p* = 0.027) (Model C). Furthermore, log 25(OH)D predicted HFrEF, maintaining its significance when corrected for age, NT-proBNP tertiles, GGT tertiles, the presence of hypertension, CCS, anemia, and hypothyroidism (*p* = 0.036) (Model D).

## 4. Discussion

Over the last few decades, especially during the COVID-19 pandemic, vitamin D received widespread attention, mainly because of its complex biological activity, regulating gene transcription, and modulating signaling pathways [31]. Vitamin D is a steroid compound and has two main forms—ergocalciferol and cholecalciferol—obtained mostly via endogenous synthesis in the skin through sunlight exposure (UVB radiation) from ergosterol and 7-dehydrocholesterol. After two sequential hydroxylation reactions, first catalyzed by hepatic 25-hydroxylases, followed by the activity of the renal 1-α hydroxylase, the biologically active 1,25(OH)_2_D is released into the bloodstream, circulating in both protein-bound (DBP, albumin) and free forms [32]. The removal of filtered DBP-25(OH)D complexes is possible in renal proximal tubules via megalin/cubilin-mediated endocytosis. Both receptors exert important roles in the physiological tubular reabsorption of albumin and other proteins [33]. Moreover, 1,25(OH)_2_D deficient mice developed glomerular (podocyte) injury, albuminuria, and subsequent kidney dysfunction, which were reversible upon exposure to 1,25(OH)_2_D [34]. Zhang et al. also highlighted the protective effects of paricalcitol, a vitamin D analogue, in type 2 diabetic (db/db) mice, which attenuated albuminuria, podocyte injury, and mesangial matrix expansion, by improving autophagic activity [35]. Our patients with HF showed significant positive correlations between total 25(OH)D and serum albumin levels. Therefore, vitamin D deficiency might be a possible contributor to hypoalbuminemia in HF by means of renal injury. Although standard HF treatment (RAAS inhibitors, sodium–glucose co-transporter-2 (SGLT2) inhibitors) improves proteinuria and limits long-term decline in kidney function [36,37], vitamin D supplementation might also help prevent and reduce the progression of chronic kidney disease (CKD) in HF. Moreover, increasing serum albumin levels during hospitalization for acute HF was associated with improved prognosis at 1-year follow-up [38].

A multiple inhibitory role regarding vitamin D in HF has been proposed: it reduces myocardial fibrosis, pathological remodeling, and atherosclerosis, by down-regulating low-grade vascular inflammation [39]. The suppression of K^+^ currents proved to be an important cause of electrophysiological remodeling in cardiac hypertrophy, which was reversed upon exposure to 10 nM of calcitriol [40]. Vitamin D depletion has been shown to increase LV mass and myocardial contractility [39]. VDRs were described in the cytosol of HL-1 rat cardiomyocytes, and their concentration increased after treatment with 1,25(OH)_2_D. Calcitriol, at concentrations above 30 nM, reduced the proliferative capacity, caused mild hypertrophy, and altered cardiomyocyte morphology [41]. The size of LV myocytes is significantly increased in VDR knockout mice compared to wild-type animals, due to the overstimulation of the RAAS [42]. VDR knockout also caused interstitial hypertrophy with increased collagen content [43]. There is an ongoing debate regarding the optimal reference range for vitamin D; 25(OH)D levels less than 20 ng/mL are considered deficient, whereas concentrations between 20 and 32 ng/mL are regarded as insufficient [44]. Other authors recommend increasing the cut-off value used to identify the lower limit of normal to 40 ng/mL [45]. Our patients had a median 25(OH)D concentration of 20.88 (IQR 15.62–28.93) ng/mL, with HFrEF showing significantly lower levels compared to HFmrEF (17.57 (IQR 15.10–28.22) ng/mL vs. 22.65 (IQR 19.51–33.79) ng/mL, *p* = 0.010). Currently, there is no guideline recommendation regarding screening for vitamin D deficiency and correct supplementation in HF. Both experimental and clinical trials linked vitamin D deficiency to the excessive activation of the RAAS with increased expression of the renin gene, higher plasma renin activity, and elevated plasma aldosterone and angiotensin II levels [1,46]. Vitamin D also acts directly on the myocardium via VDRs expressed on the surface of cardiomyocytes and fibroblasts. Both pathways are associated with adverse LV remodeling through myocyte cellular hypertrophy and myocardial fibrosis. More importantly, these changes were reversible upon restoring normal levels of vitamin D [1]. 1,25(OH)_2_D increases intracellular calcium concentrations and decreases intracardiac filling pressures, thus enhancing systolic and diastolic ventricular function [1]. According to our current findings, 25(OH)D was independently linked to LVEF in univariate and multiple regression models, maintaining its significance even after correcting for important cardiovascular confounders, such as age, NT-proBNP, the presence of CCS, hypertension, and anemia (*p* = 0.036 and *p* = 0.030; *p* = 0.027 and *p* = 0.031). Contrary to the results published by Khan et al., we found no significant relationship between 25(OH)D levels and the functional status of patients with HF, reflected by the 6MWT distance and NYHA class [3], which might be explained by the smaller sample size. Also, patients who showed low levels of 25(OH)D associated type II DM more often (10 patients vs. 4 patients, *p* = 0.061). Vitamin D deficiency was previously linked to DM by regulating inflammation and inducing insulin resistance [47]. Cassano et al. associated oxidative stress with subclinical myocardial dysfunction in hypertensive patients with normal glucose tolerance test and one-hour glycemia > 155 mg/dL [48].

Several trials addressed the potential benefits and pitfalls of vitamin D supplementation in HF but showed inconsistent results. The VINDICATE study enrolled vitamin D-deficient patients with HF and LVEF ≤ 45% already on optimal medical treatment. After 12 months of well-tolerated high-dose cholecalciferol supplementation (4000 IU daily), LV structure and function showed statistically significant improvements, reflected by the EF, end-diastolic, and end-systolic diameters, and volumes. Unexpectedly, the reversal of LV remodeling was not associated with improved 6MWT distance at 1 year, and further clinical outcomes were not assessed [49]. Another randomized placebo-controlled double-blind trial with 50,000 IU of vitamin D_3_ administered weekly and 400 mg of calcium citrate given BID for 6 months, failed to demonstrate the beneficial effects of cholecalciferol supplementation on physical performance in HF patients with a mean LVEF of 39.2 ± 13.2%, and already on guideline-directed medical therapy [50]. The more recent EVITA study included patients with end-stage HF and a median LVEF of 28%, who received 4000 IU of vitamin D_3_ daily during a 3-year follow-up. Long-term intake of moderately high doses of vitamin D had no impact on mortality but was associated with a significantly greater need for mechanical circulatory support device implants in patients with baseline circulating 25(OH)D levels ≥ 30 nmol/L, who showed increased in-study concentrations > 100 nmol/L [51]. In a cardiac surgery patient cohort, a U-shaped association was found between the preoperative vitamin D status and the risk of MACCE, using the same thresholds: <30 nmol/L and >100 nmol/L [52]. High plasma calcium might provide a potential explanation for the negative outcomes of chronic high-dose vitamin D supplementation [51]. A recently published meta-analysis on dietary interventions in HF reported improvement in LVEF and reduction in CRP levels, as a result of vitamin D intake [3]. Vitamin D supplementation also impacts the calcium regulatory molecules of the vessel wall, especially osteoprotegerin, which was reported to correlate with arterial stiffness [53,54]. Vitamin D might be associated with factors that exacerbate HF; Ernst et al. reported the association of low levels of 25(OH)D with anemia in patients with HF [55].

As seen, the trials investigating the effects of vitamin D in HF used different supplementation schemes (doses and administration intervals) and study outcomes, which might explain the inconclusive results.

Serum UA also sparked controversy regarding its status as a potential cardiovascular risk factor, considering its pro-oxidant–antioxidant activity. Elevated levels were linked to increased risk for cardiovascular disease, including HF [56]. Moreover, hyperuricemia is associated with adverse outcomes in both HFrEF and HF with preserved EF (HFpEF) [57,58]. It is important to highlight that UA has antioxidant properties capable of neutralizing free radicals [59]. Also, the increased activity of XO contributes to the inflammation and oxidative stress already present in HF [59]. Vericiguat, the oral soluble guanylate cyclase receptor stimulator, not only reduced the composite endpoint of hospitalization for HF or death from cardiovascular causes, but also significantly decreased high-sensitive CRP and serum UA concentrations in patients with HFrEF [60,61]. Our findings were concordant with the results published by Ivan et al. concerning the inverse relationship between LVEF and serum UA [62] (HFrEF vs. HFmrEF—7.43 ± 2.41 mg/dL vs. 5.69 ± 2.07 mg/dL, *p* = 0.005). We also found that patients with 25(OH)D deficiency were prone to develop hyperuricemia. The association might be explained by the severity of LV systolic dysfunction, which is associated with lower levels of vitamin D [3]; both vitamin D deficiency and hyperuricemia increase RAAS activity with subsequent adverse LV remodeling [5,23,24]. Also, these patients are more likely to benefit from loop diuretic treatment, which increases tubular urate reabsorption and decreases UA excretion [63]. Treatment with angiotensin receptor–neprilysin inhibitors (ARNI) and SGLT2 inhibitors lowered serum UA concentrations with improved outcomes in patients with HF [58,64]. The recently published ALL-HEART trial investigated the possible beneficial effects of allopurinol versus usual care in ischemic heart disease, but reported no differences in cardiovascular outcomes [59]. In patients with HF and a history of gout, treatment with allopurinol, an XO inhibitor, was tied to a significant reduction in all-cause mortality, hospitalization for HF or death [19]. The URRAH study proposed new cut-off values for UA regarding all-cause death—4.7 mg/dL—and cardiovascular mortality—5.6 mg/dL [65]. It should be noted that these serum UA concentrations are currently still regarded as within normal range.

We hypothesize that the associated changes in LVEF, 25(OH)D, serum UA, and albumin levels might indicate similar pathophysiological backgrounds in HF with systolic dysfunction. One possible explanation might rely on the pleiotropic biological impact of inflammation. Abundant evidence already linked low-grade systemic inflammation to HF [12]. Therefore, this raises the question of whether addressing inflammation using anti-inflammatory treatment strategies or targeting every parameter separately in order to reduce systemic inflammation, or both, would actually represent the optimal therapeutic strategy in HF. Our current findings are mostly hypothesis generating, and further research needs to provide missing information on possible interconnections regarding vitamin D, in order to implement a multimodal approach in HF.

## 5. Conclusions

Vitamin D, serum UA, and albumin are already established prognostic biomarkers in HF. We found 25(OH)D to be an independent marker of LVEF using univariate and multiple regression analysis. Significant correlations were identified between LVEF, 25(OH)D, serum UA, and albumin, suggesting potential molecular interactions. Although there is currently a plethora of information regarding vitamin D deficiency in HF, its precise mechanism, clinical impact, and management still need clarification.

## Figures and Tables

**Table 1:** Clinical, laboratory, and ultrasound variables in HF patients.

Characteristics	HF with Impaired EF, *n* = 70	HFrEF, *n* = 46	HFmrEF, *n* = 24	*p*-Value
*Basic characteristics, comorbidities*				
Inpatient/outpatient, no. (%)	39 (55.7)/31 (44.3)	31 (67.4)/15 (32.6)	8 (33.3)/16 (66.7)	0.019
Male/female, no. (%)	51 (72.9)/19 (27.1)	33 (71.7)/13 (28.3)	18 (75.0)/6 (25.0)	0.825
Age, mean ± SD (min; max), years	66.0 ± 10.9 (40.0; 88.0)	64.9 ± 10.9 (40.0; 88.0)	68.0 ± 10.8 (43.0; 82.0)	0.258
BMI, mean ± SD, kg/m^2^	28.6 ± 5.5	29.6 ± 5.5	26.6 ± 5.0	0.025
Heart disease, median (IQR), years	8.0 (4.0–15.0)	8.5 (5.0–15.8)	5.0 (2.0–11.3)	0.041
HF, median (IQR), years	2.0 (1.0–8.0)	3.0 (1.0–7.8)	2.0 (1.0–6.8)	0.464
NYHA class I/II/III, no. (%)	7 (10.0)/43 (61.4)/20 (28.6)	3 (6.5)/28 (60.9)/15 (32.6)	4 (16.7)/15 (62.5)/5 (20.8)	0.220
6MWT, median (IQR), m	365.5 (252.0–485.0)	320.5 (250.0–471.0)	400.0 (266.0–505.0)	0.474
MLHFQ score, median (IQR)	20.0 (10.0–40.0)	21.0 (14.0–47.0)	14.5 (4.5–25.5)	0.012
Smoking, no. (%)	17 (24.3)	14 (30.4)	3 (12.5)	0.212
Hypertension, no. (%)	38 (54.3)	22 (47.8)	16 (66.7)	0.195
Cardiomyopathy ischemic/non-ischemic, no. (%)	30 (42.9)/40 (57.1)	22 (47.8)/24 (52.2)	8 (33.3)/16 (66.7)	0.319
Mitral valve regurgitation medium/severe, no. (%)	28 (40.0)	21 (45.7)	7 (29.2)	0.256
Aortic stenosis medium/severe, no. (%)	9 (12.9)	5 (10.9)	4 (16.7)	0.689
Tricuspid regurgitation medium/severe, no. (%)	20 (28.6)	14 (30.4)	6 (25.0)	0.710
AF/AFL, no. (%)	32 (45.7)	21 (45.7)	11 (45.8)	0.995
LBBB, no. (%)	30 (42.9)	22 (47.8)	8 (33.3)	0.984
Type II DM, no. (%)	21 (30.0)	18 (39.1)	3 (12.5)	0.064
Atherosclerosis 0/1/2/3 territories *, no. (%)	34 (48.6)/14 (20.0)/15 (21.4)/7 (10.0)	20 (43.5)/11 (23.9)/8 (17.4)/7 (15.2)	14 (58.3)/3 (12.5)/7 (29.2)/0 (0.0)	0.308
Symptomatic PAD, no. (%)	9 (12.9)	7 (15.2)	2 (8.3)	0.632
CAD, no. (%)	18 (25.7)	13 (28.3)	5 (20.8)	0.609
COPD, no. (%)	12 (17.1)	9 (19.6)	3 (12.5)	0.625
Iron deficiency, no. (%)	21 (30.0)	14 (30.4)	7 (29.2)	0.935
Anemia, no. (%)	7 (10.0)	5 (10.9)	2 (8.3)	0.863
Thyroid function 0/1/2 **, no. (%)	60 (85.7)/9 (12.9)/1 (1.4)	40 (87.0)/5 (10.9)/1 (2.2)	20 (83.3)/4 (16.7)/0 (0.0)	0.804
*Ultrasound parameters*				
Lung US profile 0/1, no. (%)	53 (75.7)/17 (24.3)	32 (69.6)/14 (30.4)	21 (87.5)/3 (12.5)	0.232
LAVI, median (IQR), mL/m^2^	47.0 (37.1–65.1)	51.5 (42.5–65.4)	39.6 (29.3–56.1)	0.032
E/e’, median (IQR)	10.1 (8.1–12.8)	10.7 (8.6–13.3)	8.4 (6.8–11.5)	0.017
LVEDVI, mean ± SD, mL/m^2^	90.1 ± 31.7	100.4 ± 32.3	70.5 ± 19.0	<0.0001
LVESVI, median (IQR), mL/m^2^	51.6 (38.1–77.6)	66.2 (50.6–89.3)	35.8 (29.4–44.7)	<0.0001
LVEF, mean ± SD, %	36.0 ± 8.8	30.8 ± 5.9	46.0 ± 2.7	<0.0001
LVGLS, mean ± SD, %	−11 ± 3.5	−9.3 ± 2.6	−14.3 ± 2.5	<0.0001
SVI, mean ± SD, mL/m^2^	32.8 ± 11.1	29.4 ± 10.0	39.4 ± 10.3	0.0002
*Laboratory variables*				
WBC, mean ± SD, ×1000/µL	7.3 ± 2.1	7.4 ± 2.2	7.0 ± 1.6	0.433
Platelets, mean ± SD, ×1000/µL	238.4 ± 67.0	233.3 ± 63.8	248.0 ± 73.1	0.388
Hemoglobin, mean ± SD, g/dL	14.5 ± 2.0	14.7 ± 2.1	14.2 ± 1.6	0.266
Ferritin, median (IQR), ng/dL	119.7 (72.5–202.2)	110.1 (68.0–219.4)	131.5 (80.4–175.5)	0.867
Iron, median (IQR), μg/dL	81.5 (56.0–114.0)	74.0 (55.0–104.0)	106.0 (59.5–118.0)	0.225
Cholesterol, mean ± SD, mg/dL	171.5 ± 46.8	170.9 ± 48.1	172.5 ± 45.1	0.890
Triglycerides, median (IQR), mg/dL	111.5 (83.0–138.0)	117.5 (90.0–167.0)	96.0 (79.0–133.5)	0.158
CRP, median (IQR), mg/dL	0.3 (0.1–0.8)	0.4 (0.1–0.9)	0.3 (0.1–0.6)	0.331
Fibrinogen, mean ± SD, mg/dL	400.0 ± 127.4	416.9 ± 126.3	367.6 ± 125.7	0.125
Albumin, median (IQR), g/L	44.6 (40.1–46.3)	44.7 (41.1–46.2)	43.7 (40.0–46.9)	0.786
eGFR, mean ± SD, mL/min/m^2^	71.0 ± 21.5	67.7 ± 21.5	77.2 ± 20.4	0.079
Uric acid, mean ± SD, mg/dL	6.8 ± 2.4	7.4 ± 2.4	5.7 ± 2.1	0.005
NT-proBNP, median (IQR), pg/mL	967.3 (547.5–1890.5)	1090.4 (675.2–2664.9)	759.0 (260.3–1474.8)	0.034
25(OH)D, median (IQR), ng/mL	20.9 (15.6–28.9)	17.6 (15.1–28.2)	22.7 (19.5–33.8)	0.010
*Treatment*				
Loop diuretic, no. (%)	53 (75.7)	40 (87.0)	13 (54.2)	0.024
MRA, no. (%)	60 (85.7)	41 (89.1)	19 (79.2)	0.490
SGLT2i, no. (%)	45 (64.3)	33 (71.7)	12 (50.0)	0.135
BB, no. (%)	62 (88.6)	41 (89.1)	21 (87.5)	0.914
ACEI/ARB, no. (%)	19 (27.1)	12 (26.1)	7 (29.2)	0.835
ARNI, no. (%)	39 (55.7)	26 (56.5)	13 (54.2)	0.876
Statin and/or ezetimibe, no. (%)	49 (70.0)	32 (69.6)	17 (70.8)	0.985

BMI, body mass index; HF, heart failure; NYHA, New York Heart Association; 6MWT, six-minute walk test distance; MLHFQ, Minnesota Living with Heart Failure Questionnaire; AF, atrial fibrillation; AFL, atrial flutter; LBBB, left bundle branch block; DM, diabetes mellitus; PAD, peripheral artery disease; CAD, carotid artery disease; COPD, chronic obstructive pulmonary disease; US, ultrasound; LAVI, left atrial volume index; LVEDVI, left ventricular end-diastolic volume index; LVESVI, left ventricular end-systolic volume index; LVEF, left ventricular ejection fraction; LVGLS, left ventricular global longitudinal strain; SVI, stroke volume index; WBC, white blood cells; CRP, C-reactive protein; eGFR, estimated glomerular filtration rate; NT-proBNP, N-terminal pro B-type natriuretic peptide; 25(OH)D, 25-hydroxivitamin D; MRA, mineralocorticoid receptor antagonist; SGLT2i, sodium-glucose cotransporter-2 inhibitor; BB, beta-blocker; ACEI, angiotensin-converting enzyme inhibitor; ARB, angiotensin II receptor blocker; ARNI, angiotensin receptor/neprilysin inhibitor. * Coronary artery disease, symptomatic and asymptomatic peripheral artery disease, carotid artery disease; ** 0—normal thyroid function, 1—hypothyroidism, 2—hyperthyroidism.

**Table 2:** Correlations for LVEF in the overall HF population.

Characteristics	Overall Group		Characteristics	Overall Group	
	Spearman’s R	*p*-Value		Spearman’s R	*p*-Value
LOS, days	−0.34	0.003	WBC, ×1000/µL	−0.10	0.918
Age, years	0.18	0.125	NLR	−0.035	0.771
BMI, kg/m^2^	−0.32	0.006	Platelets, ×1000/µL	0.13	0.279
Duration heart disease, years	−0.21	0.076	MPV, fL	−0.21	0.068
Duration HF, years	−0.11	0.338	PDW, %	−0.18	0.123
SBP, mm Hg	0.35	0.003	Hemoglobin, g/dL	−0.24	0.037
DBP, mm Hg	0.32	0.006	Uric acid, mg/dL	−0.31	0.009
HR, bpm	−0.11	0.360	Albumin, g/dL	0.11	0.374
Hypertension, y/n	0.22	0.062	Cholesterol, mg/dL	0.16	0.173
ABI	0.19	0.107	HDL-cholesterol, mg/dL	0.22	0.066
6MWT distance, m	0.20	0.095	Triglycerides, mg/dL	−0.10	0.391
LAVI, mL/m^2^	−0.22	0.060	GGT, IU/L	−0.32	0.006
E/e’	−0.24	0.037	Ferritin, ng/dL	0.02	0.854
LVEDVI, mL/m^2^	−0.59	<0.0001	Iron, µg/dL	0.14	0.228
LVESVI, mL/m^2^	−0.76	<0.0001	eGFR, mL/min/m^2^	0.20	0.090
LVGLS, %	−0.82	<0.0001	Fibrinogen, mg/dL	−0.07	0.514
NT-proBNP, pg/mL	−0.26	0.029	ESR, mm/h	0.04	0.711
25(OH)D, ng/mL	0.31	0.008	TSH, µU/mL	−0.24	0.040

LOS, length of stay; BMI, body mass index; HF, heart failure; SBP, systolic blood pressure; DBP, diastolic blood pressure; HR, heart rate; ABI, ankle-brachial index; 6MWT, six-minute walk test; LAVI, left atrial volume index; LVEDVI, left ventricular end-diastolic volume index; LVESVI, left ventricular end-systolic volume index; LVGLS, left ventricular global longitudinal strain; NT-proBNP, N-terminal pro-B-type natriuretic peptide; 25(OH)D, 25-hydroxyvitamin D; WBC, white blood cells; NLR, neutrophil-to-lymphocyte ratio; MPV, mean platelet volume; PDW, platelet distribution width; HDL, high-density lipoprotein; GGT, gamma-glutamyl transferase; eGFR, estimated glomerular filtration rate; ESR, erythrocyte sedimentation rate; TSH, thyroid-stimulating hormone.

**Table 3:** Model A and Model B—multivariate linear regression analysis of the factors correlated with LVEF in the overall patient group.

Model A: Summary of Regression				
Variables	B	SE of B	β	*p*-Value
Age, years	0.129	0.090	0.159	0.155
BMI, kg/m^2^	−0.430	0.110	−0.267	0.019
Hypertension, y/n	3.831	1.996	0.218	0.060
Albumin, g/dL	−0.135	0.277	−0.063	0.628
GGT, IU/L	−0.041	0.015	−0.298	0.008
NT-proBNP, pg/mL	−0.0006	0.0003	−0.224	0.064
TSH, µU/mL	−0.050	0.154	−0.034	0.746
Log 25(OH)D	11.547	5.399	0.250	0.036
**Model B: Summary of Regression**				
**Variables**	**B**	**SE of B**	**Β**	***p*-Value**
BMI, kg/m^2^	−0.496	0.178	−0.308	0.007
Hypertension, y/n	3.224	1.886	0.184	0.092
Log 25(OH)D	10.986	4.938	0.238	0.030
NT-proBNP, pg/mL	−0.0007	0.0003	−0.264	0.023
Age, years	0.217	0.092	0.268	0.021
CCS, y/n	−3.368	1.926	−0.191	0.085

BMI, body mass index; GGT, gamma-glutamyl transferase; NT-proBNP, N-terminal pro-B-type natriuretic peptide; TSH, thyroid stimulating hormone; log 25(OH)D, log-transformed 25-hydroxivitamin D; CCS, chronic coronary syndrome.

**Table 4:** Model C and Model D—summary of the multiple logistic regression analysis of the factors predicting HFrEF in the overall patient cohort.

Model C: Summary of Regression		
Variables	OR (95% CI)	*p*-Value
BMI (kg/m^2^), tertile 3:tertile 1	3.80 (1.06–13.52)	0.113
Hypertension, y/n	0.45 (0.16–1.28)	0.048
Atrial fibrillation, y/n	0.99 (0.36–2.67)	0.578
Anemia, y/n	1.34 (0.24–7.49)	0.132
Uric acid (mg/dL), tertile 3:tertile 1	3.25 (0.96–10.92)	0.156
Albumin (g/L), tertile 1:tertile 3	0.56 (0.17–1.91)	0.085
GGT (IU/L), tertile 3:tertile 1	4.50 (1.03–19.59)	0.015
Log 25(OH)D, tertile 1:tertile 3	4.02 (1.05–15.42)	0.027
**Model D: Summary of Regression**		
**Variables**	**OR (95% CI)**	***p*-Value**
Age, years	0.50 (0.18–1.38)	0.089
Hypertension, y/n	0.45 (0.16–1.28)	0.066
CCS, y/n	1.83 (0.66–5.12)	0.113
Anemia, y/n	1.34 (0.24–7.49)	0.269
NT-proBNP (pg/mL), tertile 3:tertile 1	2.75 (0.80–9.45)	0.376
Hypothyroidism, y/n	0.61 (0.14–2.52)	0.469
GGT (IU/L), tertile 3:tertile 1	4.50 (1.03–19.59)	0.050
Log 25(OH)D, tertile 1:tertile 3	4.02 (1.05–15.42)	0.036

BMI, body mass index; GGT, gamma-glutamyl transferase; log 25(OH)D, log-transformed 25-hydroxivitamin D; CCS, chronic coronary syndrome; NT-proBNP, N-terminal pro-B-type natriuretic peptide.

## Data Availability

Data spreadsheets are available as “total 25-hydroxyvitamin D is an independent marker of left ventricular ejection fraction in heart failure with reduced and mildly reduced ejection fraction”, https://doi.org/10.6084/m9.figshare.24099942 (accessed on 12 September 2023).
